# Point-of-care ultrasonography in diagnosing necrotizing fasciitis—a literature review

**DOI:** 10.1007/s40477-022-00761-5

**Published:** 2023-01-24

**Authors:** Rick Kye Gan, Antoni Sanchez Martinez, Muhammad Abdus-Syakur Abu Hasan, Rafael Castro Delgado, Pedro Arcos González

**Affiliations:** 1 Unit for Research in Emergency and Disaster, Public Health Area, Department of Medicine, University of Oviedo, Oviedo, Asturias Spain; 2 Department of Global Public Health, Karolinska Institutet, Stockholm, Sweden; 3 Emergency Medicine Department, Kulliyyah of Medicine, International Islamic University Malaysia (IIUM), Kuantan, Pahang Malaysia; 4 Emergency and Trauma Department, Sultan Ahmad Shah Medical Center, IIUM, Kuantan, Pahang Malaysia; 5 SAMU-Asturias, Asturias, Spain

**Keywords:** Necrotizing fasciitis, Necrotizing soft tissue infections, Point-of-care ultrasonography, Diagnostic ultrasonography

## Abstract

**Introduction:**

Necrotizing fasciitis (NF) is a rapidly progressive necrosis of the fascial layer with a high mortality rate. It is a life-threatening medical emergency that requires urgent treatment. Lack of skin finding in NF made diagnosis difficult and required a high clinical index of suspicion. The use of ultrasound may guide clinicians in improving diagnostic speed and accuracy, thus leading to improved management decisions and patient outcomes. This literature search aims to review the use of point-of-care ultrasonography in diagnosing necrotizing fasciitis.

**Method:**

We searched relevant electronic databases, including PUBMED, MEDLINE, and SCOPUS, and performed a systematic review. Keywords used were “necrotizing fasciitis” or “necrotising fasciitis” or “necrotizing soft tissue infections” and “point-of-care ultrasonography” “ultrasonography” or “ultrasound”. No temporal limitation was set. An additional search was performed via google scholar, and the top 100 entry was screened.

**Results:**

Among 540 papers screened, only 21 were related to diagnosing necrotizing fasciitis using ultrasonography. The outcome includes three observational studies, 16 case reports, and two case series, covering the period from 1976 to 2022.

**Conclusion:**

Although the use of ultrasonography in diagnosing NF was published in several papers with promising results, more studies are required to investigate its diagnostic accuracy and potential to reduce time delay before surgical intervention, morbidity, and mortality.

## Introduction

Necrotizing fasciitis (NF), also known as necrotizing soft tissue infections, is defined as rapidly progressive necrosis of the fascial layer, often sparing the overlying skin and underlying muscle. NF is difficult to diagnose in its early stages due to a lack of skin findings, requiring a high clinical index of suspicion [1]. NF of the limbs accounts for a 30% mortality rate, despite new advancements in treatment and critical care management [2]. Early diagnosis of NF is vital for surgical intervention, reducing a patient's morbidity and mortality [3].

The clinical feature of NF includes fever, edema, pain, and redness with rapid deterioration into dusky-blue discoloration, with or without serosanguineous blister and crepitus. The definitive diagnosis of NF is invasive, which requires surgical exploration of the tissues [4]. Laboratories Risk Indicator for Necrotizing Fasciitis (LRINEC) may help assess a patient's risk of NF. However, LRINEC has low sensitivity and is not recommended to be used to rule out NF [5].

Advanced imaging, such as magnetic resonance imaging (MRI), is currently the gold standard for diagnosing necrotizing fasciitis, with a sensitivity of 93% [6]. Other options, such as computed tomography (CT) scan, also has high sensitivity (80%) in diagnosing NF [6]. However, MRI and CT scans are costly and not readily available in all emergency department settings. Furthermore, the use of MRI requires hours from arrangement until the results are available [7]. In addition, there is a case reported by Kehrl et al. on necrotizing fasciitis detected by ultrasonography which was missed by using CT and MRI scans [8].

Although MRI and CT scans are mainstream diagnostic modalities, point-of-care ultrasonography (POCUS) could also be handy. POCUS is defined as ultrasonography performed by the provider to obtain real-time images [9]. The nature of POCUS allows it to be easily performed and repeated anytime when required [9]. POCUS is convenient, relatively affordable, and non-invasive. POCUS will enable clinicians to obtain real-time dynamic images that could guide clinicians in diagnosing NF. The ultrasonographic findings of necrotizing fasciitis are described as STAFF—Subcutaneous irregularity or Thickening, Air and Fascial Fluid [10].

This literature search aims to review the use of point-of-care ultrasonography in diagnosing necrotizing fasciitis.

## Materials and methods

A literature review was performed to collect all the relevant publications, such as original research, reports, review, and case series concerning the use of POCUS in diagnosing NF. PubMed, MEDLINE, and SCOPUS databases were searched to locate studies that meet the objectives of this literature review. The research strategy used was: ((necrotizing fasciitis) OR (necrotising fasciitis) OR (necrotizing soft tissue infection)) AND ((point-of-care ultrasonography) OR (ultrasonography) OR (ultrasound)). No time limitation was set. An additional search was performed via Google Scholar, and the top 100 entry was screened manually.

**Fig. 1:**
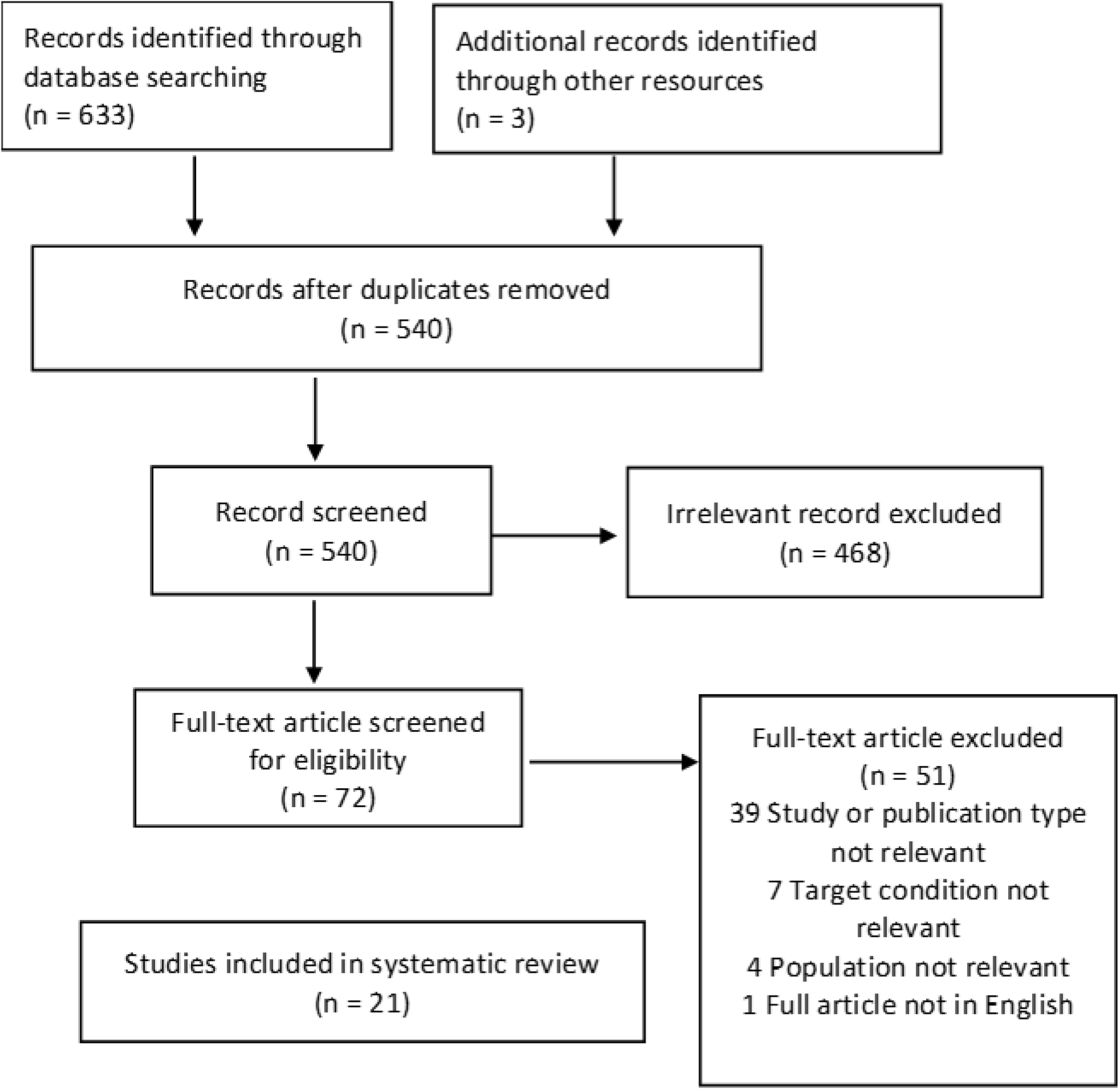
Flowchart depicting the flow of information in this literature review

The search included all articles that appeared in the literature until June 2022. First, full articles were retrieved and assessed for their suitability for review. The resulting studies were then screened initially based on their respective titles and abstracts. The flow of information in this literature review is shown in fig. 1.

The exclusion criteria were:

1. Incomplete documentation of the use of ultrasound in the diagnosis.
2. Studies focused on other diseases rather than necrotizing fasciitis.
3. Fournier’s gangrene.
4. Studies involving the pediatric population.

In the eligibility stage, the selected articles were:

1. English language reports.
2. the use of ultrasonography diagnosis for necrotizing fasciitis.

The eligible articles were extracted by two authors (the first and second) independently using predefined criteria. After the screening, the results were compared. The third reviewer resolved disharmonies between the first two authors. Next, duplicated data were removed using EndNote. The information was then extracted based on the PICO (patient, intervention, comparison, and outcome) structure. Finally, we analyzed ultrasound findings from case reports and case series using Microsoft Excel to obtain prevalence proportion and relative frequency for specific USG findings in NF. The flow of information in this literature review is shown in fig. 1.

The prevalence proportion formula is as follows:

$$\mathrm{Prevalence\,proportion}= \frac{\mathrm{The\,number\,of\,cases\,reported\,on\,specific\,USG\,findings}}{\mathrm{The\,total\,number\,of\,individual\,in\,case\,reports}}$$

The relative frequency formula is as follows:

$$\mathrm{Relative\,frequency} \left(\%\right)=\frac{\mathrm{The\,number\,of\,specific\,USG\,findings}}{\mathrm{The\,total\,number\,of\,all\,specific\,USG\,findings}} \times 100$$

## Results

Among papers concerning diagnosing necrotizing fasciitis by ultrasonography, only 21 met the inclusion criteria. We found three observational studies, 16 case reports, and 2 case series covering the period from 1976 to June 2022. The three observational studies are described in Table 1.

**Table 1:** Observational studies on emergency use of ultrasound for detection of necrotizing fasciitis

Author, year, country	Patient group	Study type	Ultrasound machine	Outcomes	Key results
Yen et al. 2002 (Taiwan) [11]	62 patients with limb infection, 17 confirmed necrotizing fasciitis	Prospective observational	Unspecified brand linear-array transducer (7.5-MHz)	Diagnostic accuracy of ultrasonography for necrotizing fasciitis	Sensitivity: 88.2%, Specificity: 93.3%, Positive predictive value: 83%, Negative predictive value: 95.4%
Lin et al. 2019, (Taiwan) [7]	95 patients with limb infection, 48 confirmed necrotizing fasciitis	Retrospective with prospective enrolment	Philips Clear Vue 550, Philips Healthcare, Bothell, WA, USA. Equipped with linear transducer (4–12 MHz)	Diagnostic accuracy of ultrasonography for necrotizing fasciitis	Sensitivity: 75%, Specificity: 70.2%, Positive Predictive value: 71.7%, Negative predictive value:72.7%, Ultrasound findings of fluid accumulation of 2 mm in fascia have the best accuracy of 72.7%, as shown in Fig. 2
Lahham et al. 2022 (USA) [12]	64 patients with soft-tissue infections	Prospective observational	Mindray TE7 ultrasound machine with a linear transducer (5–16 MHz)	Diagnostic accuracy of ultrasonography for necrotizing fasciitis	Sensitivity:100%, Specificity:98.2% Positive predictive value:88.9% Negative predictive value:100%

**Fig. 2:**
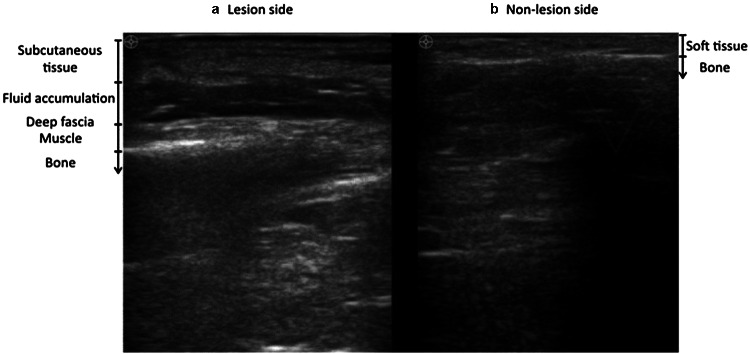
Ultrasonography shows fluid accumulation in the deep fascial layer. **a** The Lesion side had clinically suspected NF. **b** Non-lesion side had a normal skin appearance. Source: Lin et al. with permission of the author

The three observational study's location was based on the emergency department in the United States and two different emergency departments in Taiwan. All three papers adopted a convenience sampling strategy. However, studies varied in terms of the disease spectrum, prevalence, and ultrasound operator experience. All three articles utilize linear probes in performing POCUS to diagnose NF.

**Fig. 3:**
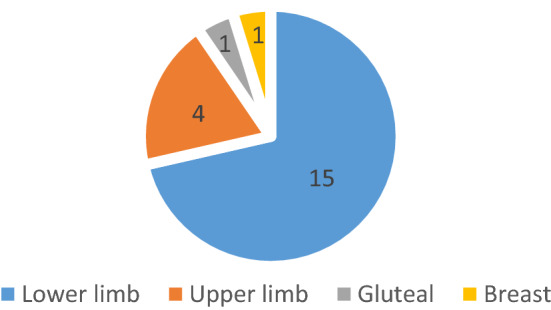
Affected anatomical location from the case report

Our literature review also revealed 17 case reports and 2 case series, which contained 5 case reports within. The age range of all patients is from 32 to 80 years old, with an average of 53 years old. In all the case reports, only a single anatomical region was involved. The most commonly affected anatomical locations were lower limbs; 15 case reports include the thigh, leg, and foot. Followed by upper limbs, 4 cases were reported, 1 case reported on the gluteal region and another one on the breast (Fig. 3, Table 2).

**Table 2:** Ultrasound findings in the case reports, their prevalence proportion and relative frequency

Specific ultrasonographic (USG) findings	Number of case reported		Prevalence proportion	Relative frequency (%)
	Yes	No		
Subcutaneous thickening	13	8	0.62	25.5
Air	13	8	0.62	25.5
Fascial fluid	17	4	0.81	33.3
Fascial irregularity	2	19	0.10	3.92
Fascial thickening	2	19	0.10	3.92
Fluid collections around tendons	1	20	0.05	1.96
Fluid collections around femur	1	20	0.05	1.96
Reduced vascularisation via doppler	1	20	0.05	1.96
Hypo-anechoic small area with blurred contours and marked edge shadowing	1	20	0.05	1.96

**Fig. 4:**
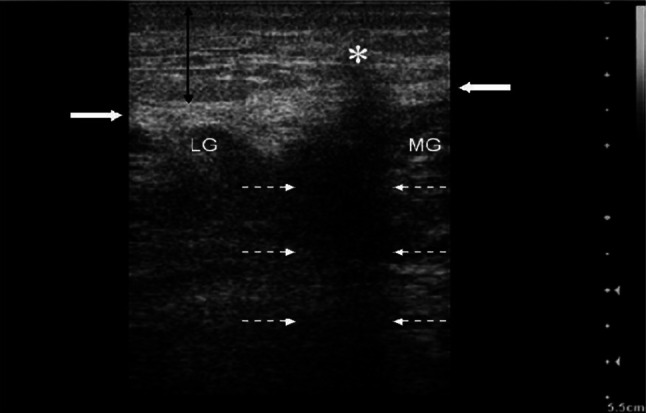
Bedside ultrasonography on the left calf with necrotizing fasciitis. Anechoic area in superficial soft tissue (*), caused by fat necrosis and suppurative infiltration, with posterior shadowing (broken opposing white arrows), which seems to break the underlying fascia. Subcutaneous tissue appears thick, and hyperechoic due to diffuse inflammatory infiltrate (vertical arrow)—source: Testa et al. with permission

All 16 case reports and 2 case series with 5 cases within described the use of ultrasound to screen for necrotizing fasciitis. Amongst the ultrasound findings, 13 of the case reported subcutaneous thickening and air. In addition, 17 of the case reported fascial fluid, and 2 of the case reported fascial irregularity. Other than that, 2 of the case reported fascial thickening, 1 case reported fluid collections around tendons, 1 case reported fluid collection around femurs, 1 case reported reduced vascularization via doppler, and 1 case reported hypo-anechoic small area with blurred contours, with marked edge shadowing which suggesting fat necrosis (Fig. 4).

The literature review revealed the most common ultrasound probe used for the purpose of diagnosing NF is a linear probe. In addition, 12 out of 21 case reports screened mentioned the use of linear probes in their diagnosis.

The most common ultrasonographic findings in NF were fascial fluid, with 33.33% of relative frequency and 0.81 prevalence proportion from the case reports analysis. Regarding the amount of fascial fluid, 2 mm depth has the best accuracy of 72.7%, 75% sensitivity, 70.2% specificity, and a positive and negative predictive value of 71.7% and 72.7%, respectively [7].

There were subcutaneous thickening findings in 13 out of 21 of the case reports screened. Moreover, Yen et al. (2002) found subcutaneous thickening accompanied by fascial fluid of 4 mm via ultrasonography yielded a sensitivity of 88.2%, 93.3% specificity, positive-predictive value: of 83.3%, and negative-predictive value of 95.4%.

**Fig. 5:**
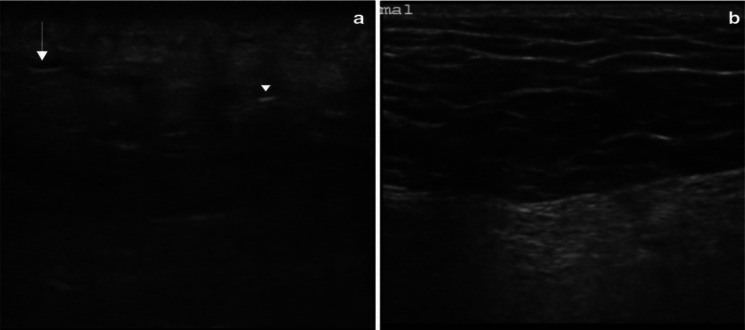
Ultrasonography of left gluteal soft tissue with **a** necrotizing fasciitis and **b** normal soft tissue on the contralateral side using a linear probe. In the affected tissue, the normal subcutaneous architecture is lost, and diffusely increased echogenicity is present. These hypoechoic regions correspond to little fluid accumulations (arrow) and a hyperechoic focus with posterior dirty acoustic shadowing, corresponding to gas in the soft tissue (arrowhead)—source: Magalhaes et al. with permission

In relation to air/subcutaneous emphysema, no article was published mentioning the sensitivity and specificity of its finding in ultrasonography of necrotizing fasciitis patients. Among our case reports, 13 out of 21 cases reported air in their ultrasonographic finding, giving rise to a prevalence proportion of 0.62 and relative frequency of 25.5%. Interestingly, Butcher et al. [13], the cadaver ultrasonography test for subcutaneous emphysema yielded a sensitivity of 100% and specificity of 87.5%. In Lin et al. (2019), 3 out of 48 patients with necrotizing fasciitis had air/subcutaneous emphysema from the ultrasonographic findings. Even Though subcutaneous emphysema is pathognomonic for necrotizing fasciitis, it is often presented in very late stages where the air accumulation is significant enough to be visible (Fig. 5). Thus, their absence should not exclude the diagnosis and cause a delay in referral to related specialty and treatment [14] (Table 3).

**Table 3:** Summary of case reports and case series

References	Age (years)	Comorbidity	Ultrasound probe	Affected site	Ultrasonographic findings	Treatment	Outcome	Remarks
Hanif 2008 [15]	34	Overweight	Linear probe	Left breast	Subcutaneous thickening (diffuse edema of the glandular tissue of the breast) Air Fascia fluid	I.V. Antibiotics Mastectomy with repeated surgical debridement	Recovered	Group A *streptococcus* infection
Hosek 2009 [16]	32	Depression and Opiate withdrawal	N/A	Left forearm pain	Air	I.V. Antibiotics Left forearm fasciotomy with debridement	Recovered	–
Wronski 2011 [17]	62	Diabetes mellitus admitted for G.I. bleeding	Linear probe	Right forearm, 5 cm above venipuncture site	Subcutaneous thickening, Air Fascial fluid (extending beyond the margins of grossly changed skin)	I.V. Antibiotics; Urgent surgical debridement	N/A	–
Testa 2013 [18]	65	Stage 4 colorectal cancer	Linear probe	Left calf	Subcutaneous thickening Fascial fluid Hypo-anechoic small area with blurred contours and marked edge shadowing (due to fat necrosis and suppurative infiltration, with posterior shadowing)	I.V. Antibiotics Surgical debridement Chemical amputation with the antiseptic alcohol solution	Discharged to family care	*E. coli* infection
Castleberg 2014 [10]	44	Morbid Obesity	N/A	Left groin and inner thigh	Subcutaneous thickening Air Fascial fluid	I.V. Antibiotics; Surgical debridement; Vacuum-assisted closure	Fully ambulatory and discharged home on postoperative day 28	Requires five days of ICU care for septic shock
Bernardi 2014 [19]	68	Nil	Linear probe	Left knee	Air	I.V. Antibiotics, fasciotomy, surgical debridement, and amputation with disarticulation of the left lower limb	Succumb to illness on day 13 of admission	–
Kehrl 2014 [8]	54	Type 2 Diabetes, peripheral neuropathy, hypertension, hyperlipidemia, tobacco abuse, obesity, depression, previous MRSA	Linear probe	Right foot and lower leg	Diffuse fascial thickening Irregularity of fascia fascial fluid fluid collection around extensor tendons	I.V. Antibiotics, incision, and debridement, admitted to ICU requiring ventilator and vasopressors	Recovered well and was discharged on day 34	*Streptococcus Pyogenes* infection
Thom 2016 [20]	78	Diabetes and peripheral arterial disease	Linear probe	Right leg	Subcutaneous thickening Air	I.V. Antibiotics, Emergency amputation	N/A	–
Clark 2017 [6]	53	Hypertension and Tachycardia	Linear probe	Left thigh	Subcutaneous thickening Air Fascial fluid	I.V. Antibiotics, Extensive debridement	N/A	Transferred to another facility
Alonso 2017 [21]	60	N/A	N/A	Left thigh	Subcutaneous thickening Air, Fascial fluid	I.V. Antibiotics, Intensive care, and high dose of vasopressor	Patients succumb to illness 24 h later	–
Martínez-Doménech 2019 [22]	50	N/a	N/A	Right forearm	Subcutaneous thickening, Fascial fluid Doppler shows reduced vascularization	I.V. Antibiotics, Decompressive fasciotomy, and tissue debridement	Recovered	Caused by *Loxosceles Rufescens* spider bite
Magalhaes 2020 [23]	54	Nil	Linear probe	Left gluteal region	Subcutaneous thickening Air Fascial fluid	I.V. Antibiotics, surgical debridement	Recovered	Initial CT-Scan was not done due to logistic
Effron 2020 [24]	32	HIV, type I diabetes	N/A	Left medial ankle (Chronic ulcer from incision and drainage 45 days ago)	Air Fascial fluid (extending from the wound up to the knee)	I.V. Antibiotics Emergency surgical debridement and osteotomy of medial malleolus Knee amputation	Discharged to skilled nursing facilities. The amputation healed well	Group C *Streptococcus, Proteus mirabilis Streptococcus mitis*
Tung-Chen 2020 [25]	68	Low back pain	N/A	Left lower limb	Subcutaneous thickening (cobblestone appearance) Fluid collection around the femur	I.V. Antibiotics, Urgent surgery	Recovered	*Streptococcus intermedius*
Fozard 2020 [26]	80	Diabetes, hypertension	Linear probe	Left lower limb	Subcutaneous thickening Superficial fascial fluid Anechoic pockets of fluid in the deep fascia	I.V. Antibiotics emergency surgery Below knee amputation and extensive debridement	Patient passed away on day 4	Group A *streptococcus* CT-scan without contrast
Martinez 2020 [27]	28	Polysubstance abuse	N/A	Left lower extremity	Subcutaneous thickening Air Fascial fluid	I.V Antibiotics, surgical operation	N/A	–
Oelze 2013 (case series) [28]	36	Diabetes type 2, hypertension, and obesity	Linear probe	Left thigh	Abscess Fascial thickening Fascial fluid Air	I.V. Antibiotics, Wide local debridement	Recovered and discharged on day 11	–
	58	nil	Linear probe	Left lower leg	Fascial thickening Fascial fluid Irregular fascial (extending proximally to the area without skin changes)	I.V. Antibiotics, Left below knee amputation	Recovered and discharged after 10 weeks	*Streptococcus pyogenes*, *Stenotrophomonas maltophilia*
	55	Hypertension, chronic kidney disease, hepatitis C, polysubstance abuse, coronary artery disease, chronic obstructive pulmonary disease	Linear probe	Right hip to the anterior thigh	Distorted edematous muscle Fascial fluid	I.V. Antibiotics, Wide debridement	Transferred to long-term acute care facility for further rehabilitation	Necrotizing fasciitis was confirmed intraoperatively. *Streptococcus pyrogen*
Shyy 2016 (case series) [29]	47	IV drug user	N/A	The left upper arm (after injecting heroin)	Subcutaneous thickening Air Fascial fluids	I.V. Antibiotics, surgical debridement	Discharged to the skilled nursing facility	Polymicrobial flora
	50	N/A	N/A	Right thigh	Fascial fluids	I.V. Antibiotics, surgical debridement	Recovered and discharged on day 11	Group A *streptococcus*

## Discussion

The literature review identified many research articles, case reports, and case series that illustrate the usefulness of POCUS in helping physicians establish the diagnosis of NF. NF is a life-threatening medical emergency that requires urgent treatment. Any delay in the treatment of NF is associated with increased morbidity and mortality [18]. POCUS is non-invasive, does not require intravenous contrast, and is free of ionizing radiation. It is also readily available in the emergency department, especially under limited resources settings.

Fozard et al. 2020 [30], recommend Sonographic exploration for fascial exploration (SEFE) examination as a systematic evaluation of the extremities for necrotizing fasciitis. However, the same principles may also apply to other body parts.

SEFE examinations include 4 steps as followings:

*Step 1*: Scan all fascial compartments using a linear transducer (i.e., anterior, lateral, superficial posterior, and deep posterior in the lower extremity) even if there are no obvious skin changes.

*Step 2*: The presence of BOTH diffuse subcutaneous thickening AND fascial fluid more than 2 mm is the diagnostic of necrotizing fasciitis.

*Step 3*: Look for supporting findings such as subcutaneous air or abnormal muscle tissue structure.

*Step 4*: Mark the area of US findings on the patient skin and consult surgery for exploration.

Castleberg et al. [10], recommend looking for Subcutaneous Thickening, Air and Fascial fluid (STAFF) examination through ultrasonography for suspected cases of NF. A combination of SEFE examination and STAFF examination is a systematic way to diagnose necrotizing fasciitis using point-of-care ultrasonography in emergency and limited-resource settings.

**Fig. 6:**
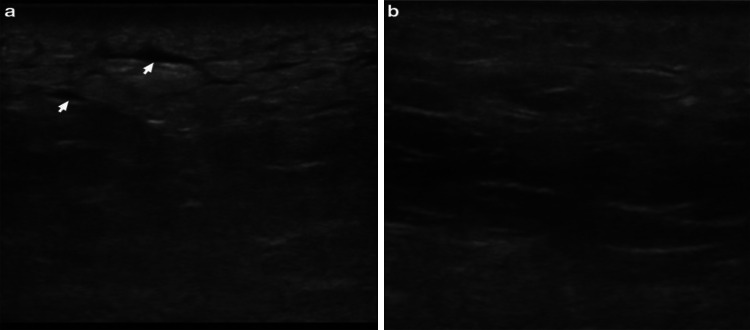
Ultrasonography of left gluteal soft tissue with **a** necrotizing fasciitis four days post-hospitalization and **b** 8 days post-hospitalization using a linear probe. These images show the progression of the sonographic appearance of necrotizing fasciitis, initially with an accumulation of fluid in the subcutaneous tissue (arrows), giving it a “cobblestone” appearance and a progressive return to the normal architecture of the subcutaneous tissue over time. Source: Magalhaes et al. with permission

POCUS has also been reported to diagnose non-infective necrotizing fasciitis. Martínez-Doménech et al. [22] described the use of POCUS in NF caused by the envenomation of *the Loxosceles rufescens* spider, which has necrotoxin property [31]. Suggest the possibility of using POCUS in monitoring the progress of envenomation and diagnosis of non-infective NF. Figure 6 shows the ultrasonographic evolution of necrotizing fasciitis recovering over time.

**Fig. 7:**
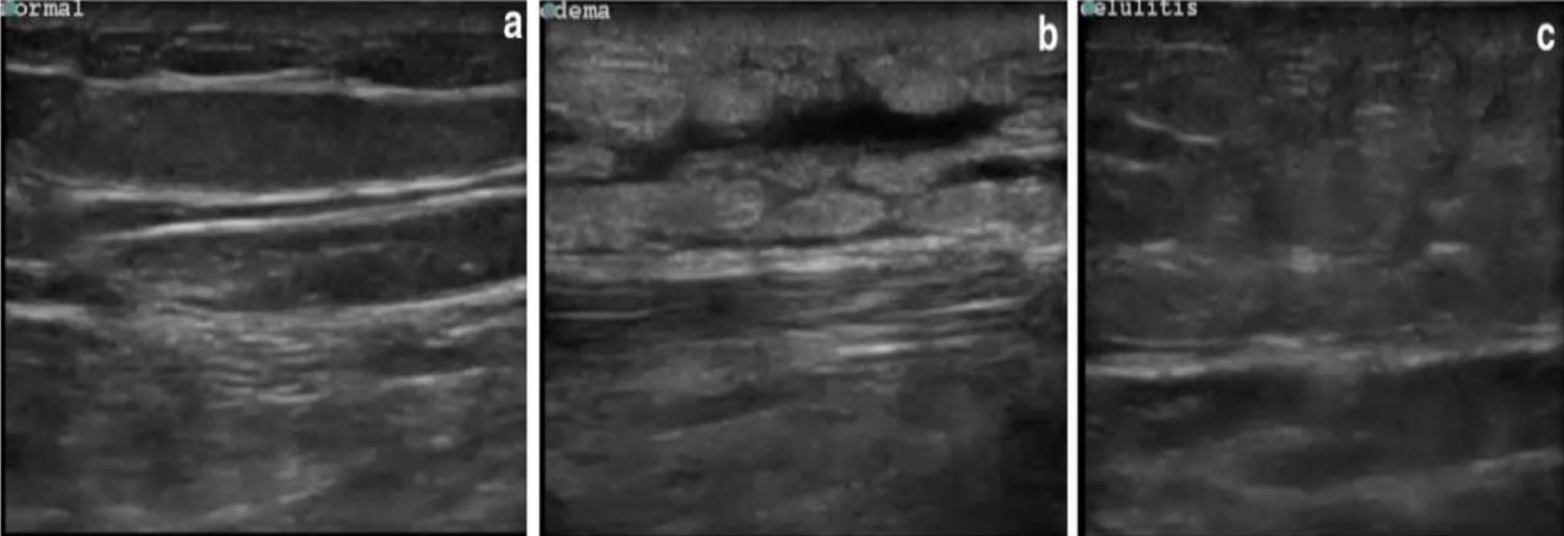
Differential diagnosis to be considered from ultrasonography findings of subcutaneous thickening and fascial fluid. **a** Normal soft tissue, **b** peripheral edema, and **c** cellulitis using a linear probe. Source: Magalhaes et al. with permission

The differential diagnosis to be considered for subcutaneous thickening and fascial fluid will be cellulitis and other causes of soft tissue edema, such as simple stasis edema, as shown in Fig. 7. For soft tissue air collection, differential diagnosis of gas-related trauma or iatrogenic causes should be considered, especially in patients with a history of surgery or trauma who did not show symptoms of sepsis. Deep vein thrombosis, foreign bodies, and abscesses could also be assessed by using ultrasonography.

High-frequency ultrasound probes greater than 8 MHz are recommended for skin and soft tissue ultrasound because they allow for good image resolution when scanning superficial structures. In addition, linear ultrasound probes enable optimum contact with the skin, and ample ultrasound gel will decrease the amount of air between the transducer and the patient’s skin, resulting in an optimum image [32].

Hence, the summary of Ultrasonography findings in diagnosing Necrotizing fasciitis is as follows [33]:

1. Subcutaneous thickening.
2. Air collection.
3. Fascial fluid.
4. Irregularity thickening of fascia.
5. Role in assessing for deep vein thrombosis, foreign bodies, and abscesses.
6. Role in monitoring the progression of necrotizing fasciitis.

## Limitation

This study also has several limitations. There are disadvantages to the usage of POCUS as a diagnostic tool for NF. The findings and quality of the examination are operator-dependent. POCUS examination also provides a limited field of vision and is not suitable for large anatomical areas of study. In the case of an obese or very muscular patient, tissue resolution may be compromised for deeper tissue depth visualization [34].

The availability of data published on the diagnostic accuracy of POCUS in the diagnosis of NF is limited. More robust quality data are needed for the purpose of meta-analysis. The case report available mainly focuses on NF of extremity, except for 2 case reports describing NF on breast and gluteal, again reinforcing the difficulty of POCUS in large anatomical areas of study.

## Conclusion

Although the use of ultrasonography in diagnosing necrotizing fasciitis was published in several papers with promising results, more studies are required to investigate its diagnostic accuracy and potential to reduce time delay before surgical intervention, morbidity, and mortality.
